# Communal nesting shapes the sex-dependent glutamatergic response to early life stress in the rat prefrontal cortex

**DOI:** 10.3389/fpsyt.2024.1406687

**Published:** 2024-05-21

**Authors:** Francesca Mottarlini, Beatrice Rizzi, Giorgia Targa, Valeria Buzzelli, Melania Di Trapano, Laura Rullo, Sanzio Candeletti, Roberto Ciccocioppo, Liana Fattore, Patrizia Romualdi, Fabio Fumagalli, Viviana Trezza, Lucia Caffino

**Affiliations:** ^1^ Department of Pharmacological and Biomolecular Sciences ‘Rodolfo Paoletti’, Università degli Studi di Milano, Milan, Italy; ^2^ Center for Neuroscience, University of Camerino, Camerino, Italy; ^3^ Department of Science, Section of Biomedical Sciences and Technologies, Roma Tre University, Rome, Italy; ^4^ Department of Pharmacy and Biotechnology, University of Bologna, Bologna, Italy; ^5^ School of Pharmacy, Center for Neuroscience, Pharmacology Unit, University of Camerino, Camerino, Italy; ^6^ Research National Council (CNR) Institute of Neuroscience-Cagliari, National Research Council, Cagliari, Italy; ^7^ Neuroendocrinology, Metabolism and Neuropharmacology Unit, Istituto di Ricerca e Cura di Carattere Scientifico (IRCCS) Fondazione Santa Lucia, Rome, Italy

**Keywords:** communal nesting, early-life stress, social isolation, NMDA receptors, prefrontal cortex, sex difference

## Abstract

**Introduction:**

Early social environment, either positive or negative, shapes the adult brain. Communal nesting (CN), a naturalistic setting in which 2-3 females keep their pups in a single nest sharing care-giving behavior, provides high level of peer interaction for pups. Early social isolation (ESI) from dam and siblings represents, instead, an adverse condition providing no peer interaction.

**Methods:**

We investigated whether CN (enrichment setting) might influence the response to ESI (impoverishment setting) in terms of social behavior and glutamate system in the medial prefrontal cortex (mPFC) of adult and adolescent male and female rats.

**Results:**

Pinning (a rewarding component of social play behavior) was significantly more pronounced in males than in females exposed to the combination of CN and ESI. CN sensitized the glutamate synapse in the mPFC of ESI-exposed male, but not female, rats. Accordingly, we observed (i) a potentiation of the glutamatergic neurotransmission in the mPFC of both adolescent and adult males, as shown by the recruitment of NMDA receptor subunits together with increased expression/activation of PSD95, SynCAM 1, Synapsin I and αCaMKII; (ii) a de-recruiting of NMDA receptors from active synaptic zones of same-age females, together with reduced expression/activation of the above-mentioned proteins, which might reduce the glutamate transmission. Whether similar sex-dependent glutamate homeostasis modulation occurs in other brain areas remains to be elucidated.

**Discussion:**

CN and ESI interact to shape social behavior and mPFC glutamate synapse homeostasis in an age- and sex-dependent fashion, suggesting that early-life social environment may play a crucial role in regulating the risk to develop psychopathology.

## 1 Introduction

Several lines of evidence have demonstrated that early social environment shapes the adult brain (1) and lack of parental figures or poor parent-child relationships during childhood may influence brain development and result in psychiatric disorders, such as depression and anxiety (2, 3). In this context, besides parent-child interactions, peer-peer relationships during infancy and then adolescence play a key role (4, 5). From a more general point of view, the social environment to which the infant brain is exposed sculpts the neuronal network that will eventually allow the infant, once grown up, to respond adequately to external stimuli, whether negative or positive. Different early life social experiences, both positive and adverse, can be mimicked in laboratory animals thus allowing to dissect the factors that contribute to alter the trajectory of brain development (6). While numerous experimental evidence exist demonstrating how alterations in mother-pups interaction can have negative consequences on brain development (7, 8), much less studies are available that investigated the positive effect induced by an enriched social environment during the first phases of life and its consequences on brain development. Particularly, communal nesting (CN), which can be considered a naturalistic setting in which two or three females keep their pups in a single nest and share care-giving behavior, represents a condition providing high level of peer interaction for rodent pups (9–11). Thus, CN represents a suitable model to investigate how early social enrichment impacts on the adult brain and behavior, as it combines the early enrichment deriving from both mother-offspring interaction and peer interaction (12, 13). Among the different aversive situations occurring during early life, pre-weaning social isolation (ESI) is among the less characterized. Evidence exists showing the sex-dependent effect of ESI on susceptibility to drugs of abuse, primarily psychostimulants (14), impulsive behavior in adolescent rats (14), proneness to develop depressive-like behavior in response to stress (15), and on locomotion, sensorimotor gating, and compulsive-like behaviors in rats (13). Thus, environmental manipulations during a period that immediately precedes weaning, when rats prepare to survive regardless of the dam, may have significant long-term, sex-specific consequences on brain development and behavior.

Social play behavior is one of the earliest forms of non-mother-directed social behavior highly expressed by young mammals, including rats, between weaning and sexual maturation (16) and it has a crucial role for proper behavioral development (17). The medial prefrontal cortex (mPFC) has been shown to play a key role in social play behaviors in humans (4, 18, 19) and animals (18, 20). Importantly, social play behavior is modulated by the glutamatergic neurotransmission in a sex-specific manner (21, 22). At the synapse, early in life manipulations, both positive such as CN or negative such as maternal separation or social isolation, modify the homeostasis of glutamate release, the molecular composition of the glutamatergic synapse, i.e. NMDA and AMPA receptor subunits, and their structure in cortical regions changing the synaptic strength and synaptic stability in age- and sex-dependent manner (23–25). However, little is known on how the combination of positive and negative social experiences in the peri- and postnatal period might shape the cortical glutamatergic synapse later in life in males and females.

We thus decided to evaluate the impact of social manipulations early in life on social play behavior during adolescence and to undertake a comprehensive study of the glutamatergic synapse homeostasis in the mPFC. We applied a brief period of social isolation during the third postnatal week of pups reared in CN or SH housing conditions as this is an age range that in laboratory rat is characterized by the maturation of crucial social, sensory, motor and cognitive abilities (26) and in which reconfiguration of the neuronal epigenome and extensive synaptogenesis occur in the brain (27). The ability of this ESI protocol to induce physiological and emotional changes in rodents has been previously demonstrated. For instance, it has been shown that mice exposed to the same ESI protocol used in our study showed depressive-like behaviors at adulthood (28) associated with epigenetic changes in different brain regions (29). Furthermore, we have recently shown that this ESI protocol induces subtle and sex-specific behavioral changes in the rat offspring (13, 30). We investigated the expression of the glutamate NMDA receptor subunits (GluN1, GluN2A, GluN2B) (31) together with the expression of the integral protein of the glutamate synapse, postsynaptic density protein-95 (PSD95), and of SynCAM1 (Synaptic Cell Adhesion Molecule), a cell adhesion molecule with signal transmission capacity, specialized in modulating the structure of excitatory synapses. In parallel, we measured the levels of phosphorylation of Synapsin1 and αCaMKII as indexes of presynaptic neurotransmitter release. Analyses were carried out longitudinally from mid adolescence (postnatal day, PND, 35) to young adulthood (PND75) to evaluate the short- and long-term persistence of the effects caused by CN, ESI and/or their combination, and in both male and female rats to detect potential underlying sex-dependent differences.

## 2 Materials and methods

### 2.1 Animals and nesting conditions

Wistar rats (Charles River Laboratories, Italy) weighing 250 ± 15 g were mated overnight. Pregnant rats assigned to the standard housing condition (SH) group were individually housed in Macrolon cages (40×26 ×20 cm; l×w×h), while pregnant rats assigned to the CN condition were housed in groups of 3 in larger Macrolon cages (62×44×22 cm; l×w×h). Both experimental groups were kept under controlled conditions (temperature 20-21°C, 55-65% relative humidity and 12/12 h light cycle with lights on at 07:00 a.m.). Food and water were available *ad libitum*. Newborn litters found up to 17:00 were considered to be born on that day [postnatal day (PND) 0]. To avoid the litter effect, one pup per litter, from different litters per experimental group, was randomly used in each behavioral experiment. Sample size (n) was based on our previous experiments and power analysis performed with the software G*Power 3.1. The sample size for each experiment is indicated in the figure legends. The experiments were approved by the Italian Ministry of Health (authorization n. 612/2020-PR) and performed in agreement with the ARRIVE (Animals in Research: Reporting *In Vivo* Experiments) guidelines (32), the guidelines of the Italian Ministry of Health (D.Lgs. 26/14) and the European Community Directive 2010/63/EU.

### 2.2 Experimental groups

We applied a protocol of environmental manipulation in the rat based on housing the animals in either socially enriched (CN) or socially impoverished (ESI) conditions from birth to weaning (PND21), compared to standard housing (SH) conditions. Therefore, rats were subjected to one of the following environmental conditions:

#### 2.2.1 Standard housing

Male and female offspring born from rat dams mated individually with a male. After one week of mating, the female was isolated and left undisturbed until the day of birth. 24 h after birth, the litter was reduced to 8 animals (4 males and 4 females) that were left undisturbed until weaning.

#### 2.2.2 Communal nesting

Male and female offspring born from rat dams subjected to the CN procedure, i.e., 3 females were housed together with a male. The male was removed one week after mating and the 3 females were left undisturbed until birth. Twenty-four hours after birth, the progeny was randomly reduced to 24 animals (12 males and 12 females) that were left undisturbed until weaning.

#### 2.2.3 Standard housing and early social isolation

male and female offspring born from rat dams mated in the SH conditions. From PND14 to PND21, an ESI protocol was applied, in which each pup was removed from the nest and singly placed in a cage with clean bedding for 30 min/day.

#### 2.2.4 Communal nesting and early social isolation

male and female offspring born from rat dams mated as in CN conditions. From PND14 to PND21, an ESI protocol was applied, in which each pup was removed from the nest and singly placed in a cage with clean bedding for 30 min/day.

Mother-offspring interactions have been evaluated as previously described (33, 34). CN reduced maternal behavior and ultrasonic vocalizations (USV) of only male pups at PND9 (data not shown), similarly to the data obtained in our previous study (35). On PND21, pups were weaned and housed in groups belonging to the same environmental condition.

**Figure 1:**
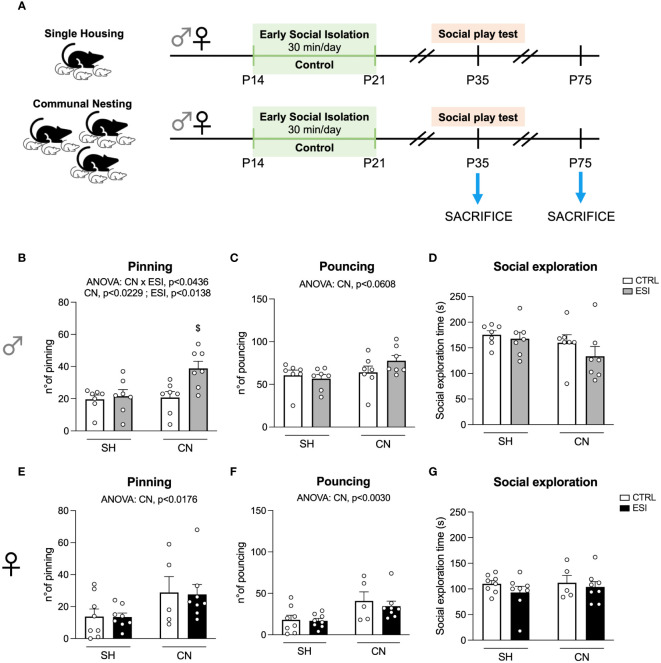
Effects of SH, CN and ESI protocol on social play behavior in PND35 male (*top*) and female (*bottom*) rats. Panel **(A)** represents a graphical timeline of the experimental paradigm in both female and male rats at PND35 and PND75. Social play behavior is expressed as number of pinning (**B** in males and **E** in females) and number of pouncing (**C** in males and **F** in females). The time spent by male **(D)** and female **(G)** rats in general social exploration is also shown. ^$^p<0.05 vs CN+CTRL (Two-way ANOVA followed by Tukey’s multiple comparisons test). SH, Standard Housing; CN, Communal Nesting; CTRL, controls - no ESI; ESI, Early Social Isolation.

The behavioral experiments were carried out in female and male offspring from all experimental groups at PND35 (males: n=7/group; females: n=8 in SH-CTRL, SH-ESI, CN-ESI, n=5 in CN-CTRL). Another cohort of female and male offspring from all experimental groups has been left undisturbed in their home-cages following the end of the ESI protocol and then sacrificed at PND35 (n=6/group per sex) or at PND75 (n=6/group per sex), to evaluate the short- or long-term impact of the early in life manipulations on the homeostasis of the glutamatergic synapse (see timeline in **Figure 1A**).

### 2.3 Behavioral test: social play

The test was performed at PND35 in both male and female offspring born in SH or CN conditions in a sound-attenuated chamber under dim light conditions, as previously described (36). The testing arena consisted of a Plexiglas cage (40×40×60 cm) with ~2 cm of wood shavings covering the floor. Rats were individually habituated to the test cage for 10 min on 2 days prior to testing. On the test day, rats were socially isolated for 3.5 h before testing. The test consisted of placing two animals into the test cage for 15 min. The animals in a test pair did not differ more than 10 g in body weight and had no previous common social experience (i.e., they were not cage mates). A pair of rats was considered as one experimental unit, and the behavioral parameters were therefore scored per pair of animals. The Observer 3.0 software (Noldus Information Technology BV, Wageningen-The Netherlands) was used to score behaviors related to play. In rats, a bout of social play behavior starts with one rat soliciting (“pouncing”) another animal by attempting to nose or rub the nape of its neck. If the animal that is pounced upon fully rotates to its dorsal surface “pinning” is the result (one animal lying with its dorsal surface on the floor with the other animal standing over it), which is considered the most characteristic posture of social play behavior in rats (37). We determined, (a) frequency of pinning, (b) frequency of pouncing and (c) time spent in social exploration (i.e., the total amount of time spent in non-playful forms of social interaction, like sniffing any part of the body of the test partner, including the anogenital area or grooming any part of the partner body).

### 2.4 Tissue collection

Rats used for molecular analyses were decapitated on PND35 and PND75 and brains were quickly removed. Brains were immediately placed into an ice-cold plate and mPFC was dissected under stereomicroscope, according with rat brain atlas (38). Tissues were stored at −80°C until analysis. These brains were collected as previously described (Rullo et al., 2023) and their processing was optimized to minimize the number of animals used in the experiment.

### 2.5 Protein extracts preparation and western blot analysis

Proteins from mPFC tissues were homogenized using a cold buffer containing 0.32M sucrose, 0.1mM PMSF, 1mM HEPES, 0.1mM EGTA pH 7.4, in presence of commercial cocktails of protease (Roche) and phosphatase (Sigma-Aldrich) inhibitors and crude membrane fraction prepared as previously described (39) and reported in the **Supplementary Material**. Total amount of proteins in the crude membrane fraction was quantified according to the Bradford Protein Assay procedure (Bio-Rad, Milan, Italy), with bovine serum albumin as calibration standard. Samples were stored at -20°C until molecular analysis.

Eight micrograms of proteins for each sample were run on a sodium dodecyl sulfate-8% polyacrylamide gel under reducing conditions and electrophoretically transferred onto nitrocellulose membrane (Bio-Rad Laboratories). Blots were blocked 1h at room temperature (RT) with I-Block solution (Life Technologies Italia) in TBS+0.1% Tween-20 buffer, incubated with antibodies against the phosphorylated forms of the proteins and then stripped in a buffer containing SDS 2%, Tris HCl pH 6.7 1M and β-mercaptoethanol and re-probed with the antibodies against corresponding total proteins. Similarly, antibodies against proteins that run at similar molecular weight were incubated first with antibodies against the less expressed protein and then stripped and re-probed with the antibodies against the more expressed proteins.

**Figure 2:**
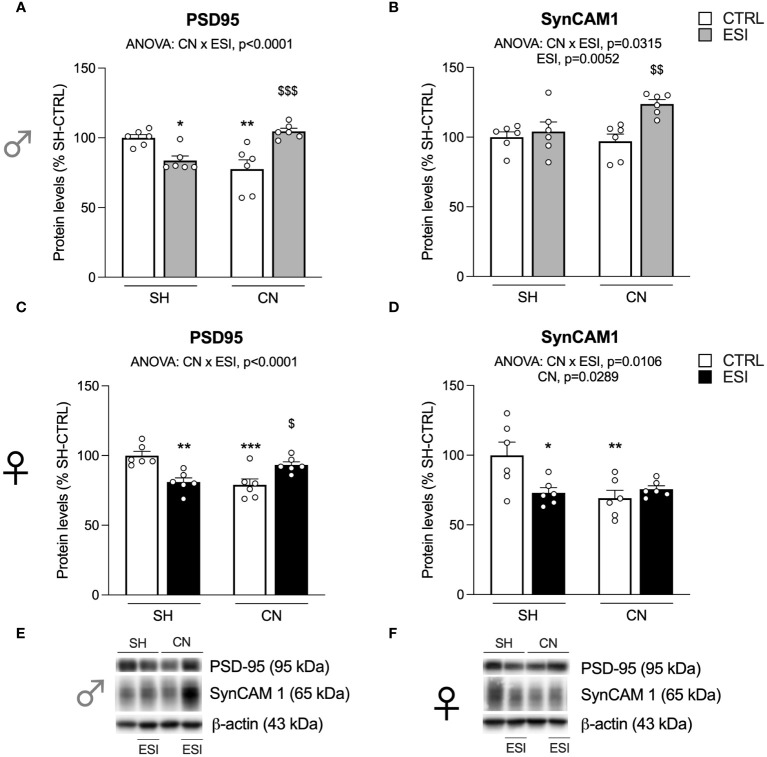
Effect of SH, CN and ESI protocol on synaptic structural markers expression in the crude membrane fraction of the mPFC of PND35 male (*top*) and female (*middle*) rats. Protein levels of PSD95 **(A)**, SynCAM 1 **(B)** in male rats and PSD95 **(C)**, SynCAM 1 **(D)** in female rats are expressed as percentages of SH-CTRL and represent the mean ± S.E.M. of six rats per group. Representative immunoblots (*bottom*) are shown for each protein in the respective panels **(E, F)**. ^*^p<0.05, ^**^p<0.01, ^***^p<0.001 vs. SH-CTRL, ^$^p<0.05, ^$$^p<0.01, ^$$$^p<0.001 vs CN+CTRL (Two-way ANOVA followed by Tukey’s multiple comparisons test). SH, Standard Housing; CN, Communal Nesting; CTRL, controls no ESI; ESI, Early Social Isolation.

**Figure 3:**
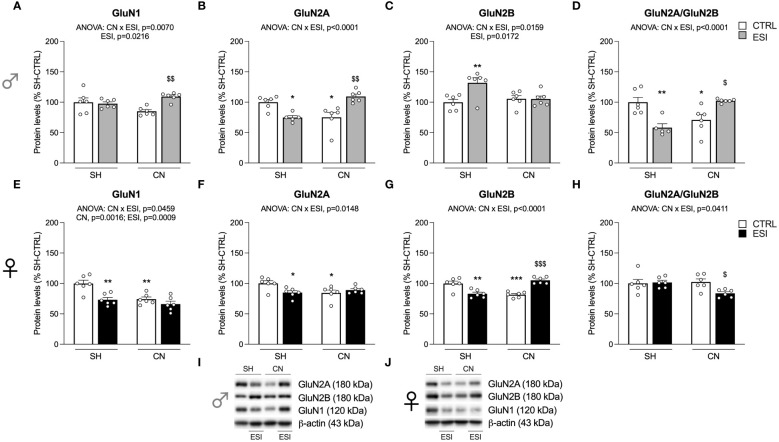
Effect of SH, CN and ESI protocol on NMDA receptor subunits in the crude membrane fraction of the mPFC of PND35 male (*top*) and female (*middle*) rats. Protein levels of GluN1 **(A)**, GluN2A **(B)**, GluN2B **(C)**, GluN2A/GluN2B ratio **(D)** in male rats, and GluN1 **(E)**, GluN2A **(F)**, GluN2B **(G)**, GluN2A/GluN2B ratio **(H)** in female rats are expressed as percentages of SH-CTRL and represent the mean±S.E.M. of six rats per group (GluN2A and GluN2A/GluN2B ratio: 1 outlier in SH-ESI males). Representative immunoblots (*bottom*) are shown for each protein in the respective panels **(I, J)**. ^*^p<0.05, ^**^p<0.01, ^***^p<0.001 vs. SH-CTRL, ^$^p<0.05, ^$$^p<0.01, ^$$$^p<0.001 vs CN+CTRL (Two-way ANOVA followed by Tukey’s multiple comparisons test). SH, Standard Housing; CN, Communal Nesting; CTRL, controls no ESI; ESI, Early Social Isolation.

**Figure 4:**
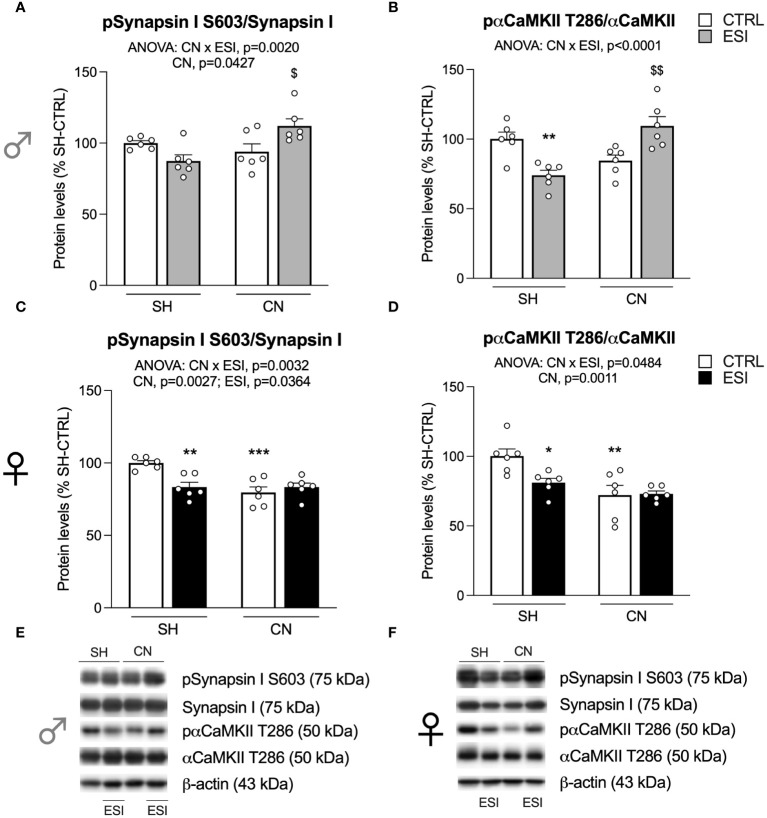
Effect of SH, CN and ESI protocol on proteins regulating neurotransmitter release in the crude membrane fraction of the mPFC of PND35 male (*top*) and female (*middle*) rats. Protein levels of pSynapsin I S603/Synapsin I **(A)**, pαCaMKII T286/αCaMKII **(B)** in male rats and pSynapsin I S603/Synapsin I **(C)**, pαCaMKII T286/αCaMKII **(D)** in female rats are expressed as percentages of SH-CTRL and represent the mean±S.E.M. of six rats per group. Representative immunoblots (*middle*) are shown for each protein in the respective panels **(E, F)**. ^*^p<0.05, ^**^p<0.01, ^***^p<0.001 vs. SH-CTRL, ^$^p<0.05, ^$$^p<0.01 vs CN+CTRL (Two-way ANOVA followed by Tukey’s multiple comparisons test). SH, Standard Housing; CN, Communal Nesting; CTRL, controls no ESI; ESI, Early Social Isolation.

**Figure 5:**
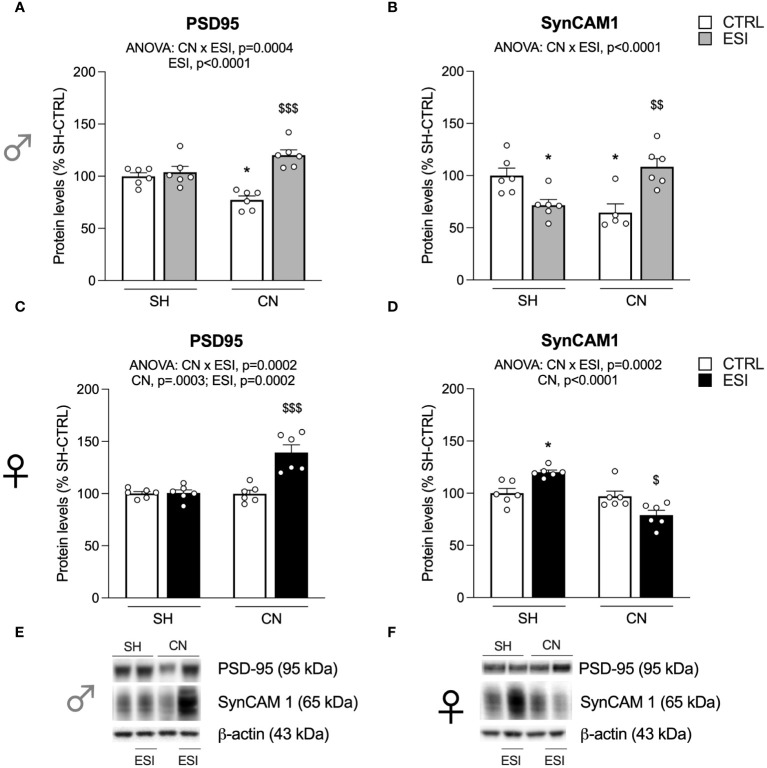
Effect of SH, CN and ESI protocol on synaptic structural markers expression in the crude membrane fraction of the mPFC of PND-75 male (*top*) and female (*bottom*) rats. Protein levels of PSD95 **(A)**, SynCAM 1 **(B)** in male rats and PSD95 **(C)**, SynCAM 1 **(D)** in female rats are expressed as percentages of SH-CTRL and represent the mean ± S.E.M. of six rats per group (SynCAM: 1 outlier in CN-CTRL males). Representative immunoblots (*bottom*) are shown for each protein in the respective panels **(E, F)**. ^*^p<0.05 vs. SH-CTRL, ^$^p<0.05, ^$$^p<0.01, ^$$$^p<0.001 vs CN+CTRL (Two-way ANOVA followed by Tukey’s multiple comparisons test). SH, Standard Housing; CN, Communal Nesting; CTRL, controls no ESI; ESI, Early Social Isolation.

**Figure 6:**
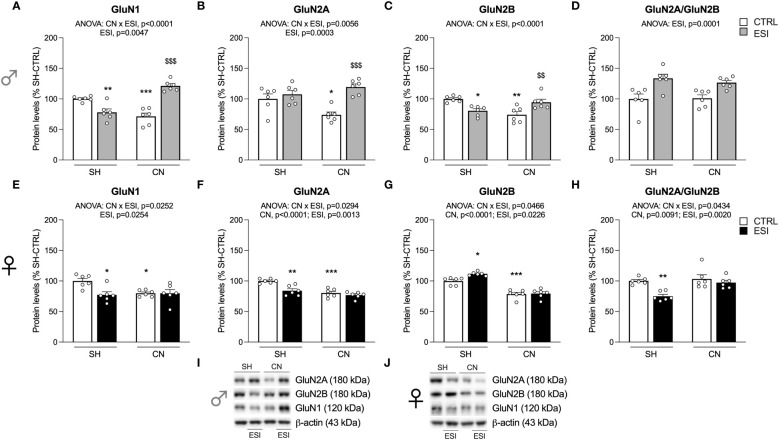
Effect of SH, CN and ESI protocol on NMDA receptor subunits in the crude membrane fraction of the mPFC of PND75 male (*top*) and female (*middle*) rats. Protein levels of GluN1 **(A)**, GluN2A **(B)**, GluN2B **(C)**, GluN2A/GluN2B ratio **(D)** in male rats and GluN1 **(E)**, GluN2A **(F)**, GluN2B **(G)**, GluN2A/GluN2B ratio **(H)** in female rats are expressed as percentages of SH-CTRL and represent the mean ± S.E.M. of six rats per group **(I, J)**. Representative immunoblots (*bottom*) are shown for each protein in the respective panels. ^*^p<0.05, ^**^p<0.01, ^***^p<0.001 vs. SH-CTRL, ^$$^p<0.01, ^$$$^p<0.001 vs CN+CTRL (Two-way ANOVA followed by Tukey’s multiple comparisons test). SH, Standard Housing; CN, Communal Nesting; CTRL, controls no ESI; ESI, Early Social Isolation.

**Figure 7:**
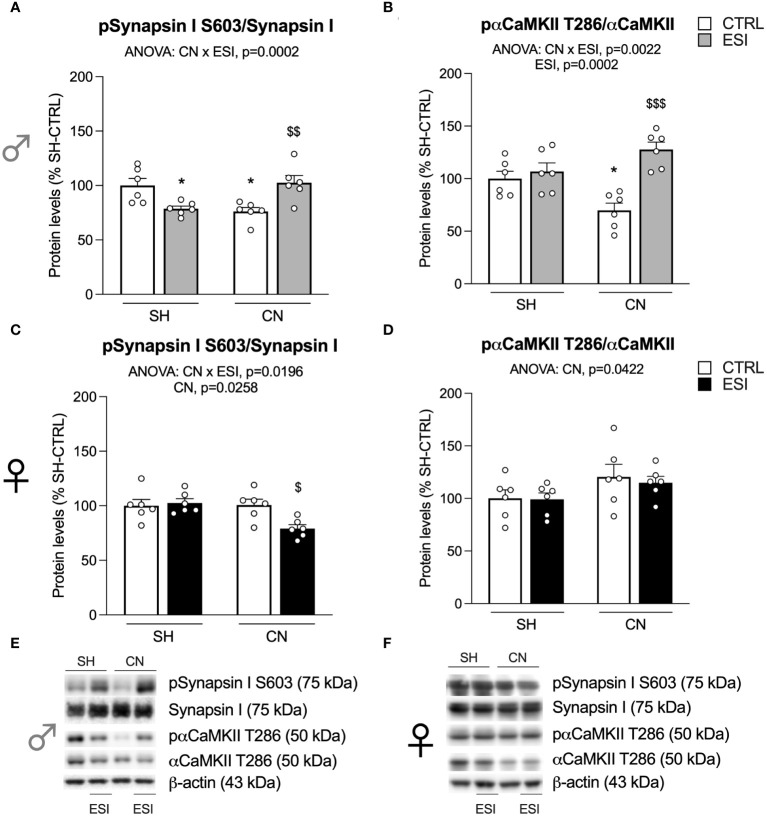
Effect of SH, CN and ESI protocol on proteins regulating neurotransmitter release in the crude membrane fraction of the mPFC of PND75 male (*top*) and female (*middle*) rats. Protein levels of pSynapsin I S603/Synapsin I **(A)**, pαCaMKII T286/αCaMKII **(B)** in male rats and pSynapsin I S603/Synapsin I **(C)**, pαCaMKII T286/αCaMKII **(D)** in female rats are expressed as percentages of SH-CTRL and represent the mean±S.E.M. of six rats per group. Representative immunoblots (*bottom*) are shown for each protein in the respective panels **(E, F)**. ^*^p<0.05 vs. SH-CTRL, ^$^p<0.05, ^$$^p<0.01, ^$$$^p<0.001 vs CN+CTRL (Two-way ANOVA followed by Tukey’s multiple comparisons test). SH, Standard Housing; CN, Communal Nesting; CTRL, controls no ESI; ESI, Early Social Isolation.

Results were standardized to β-actin control protein detected at 43kDa. Immunocomplexes were visualized by chemiluminescence using the Chemidoc MP Imaging System (Bio-Rad Laboratories) and analyzed with Image LabTM software (Bio-Rad) by evaluating the band density and representative immunoblots for each protein are shown in **Figures 2E, F**, **3I, J**, **4E, F**, **5E, F**, **6I, J**, **7E, F**. As gels were run in duplicate, the results from the two gels were averaged with a correction factor: correction factor gel B=average of (OD protein of interest/OD β-actin for each sample loaded in gel A)/(OD protein of interest/OD β-actin for the same sample loaded in gel B) (40).

The conditions of the primary antibodies were the following: anti GluN1 (1:1000, Cell Signaling Technology Inc., cod. 5704, RRID: AB_1904067), anti GluN2B (1:1000, Cell Signaling Technology Inc., cod. 14544, RRID: AB_2798506), anti GluN2A (1:1000, Cell Signaling Technology Inc., cod. 4205, RRID: AB_2112295), anti PSD95 (1:2000, Cell Signaling Technology Inc., cod. 3450, RRID: AB_2292883), anti phospho-Synapsin I Ser603 (1:1000, Cell Signaling Technology Inc., cod. 88246, RRID: AB_2800119), anti Synapsin I (1:2000, Santa Cruz Biotechnology, cod. sc-8295, RRID: AB_677472), anti phospho-αCaMKII Thr286 (1:1000, Termo Fisher Scientific, cod. MA1047, RRID: AB_325402), anti αCaMKII (1:2000, Millipore, cod. 05-532, RRID: AB_309787), anti SynCAM 1 (1:1000, Novus Biologicals, cod. NB300-186, RRID: AB_2121874), and anti β-actin (1:5000, Sigma-Aldrich, cod. A5441, RRID: AB_476744).

### 2.6 Statistical analysis

All animals tested were treated as independent values, there were no technical replicates. The molecular and behavioral changes produced by CN and ESI, alone or in combination, were analyzed using a two-way ANOVA, with the factors CN and ESI as independent variables. When dictated by relevant interaction terms, Tukey’s multiple comparisons test was used to characterize differences among individual groups of rats. Two-way ANOVA analyses were performed using raw data (**Supplementary Table 1** for the behavioral analysis, **Supplementary Table 2** for the molecular analysis performed at PND35, **Supplementary Table 3** for the molecular analysis performed at PND75). Then, data were normalized as percentages of the standardly housed (SH) control rats that were not exposed to either CN or ESI to enable visual comparisons across environmental conditions with different degrees of expression of glutamatergic molecular determinants. Subjects were eliminated from the final dataset if their data deviated from the mean by 2 SDs. Prism 8.0 (GraphPad) was used to analyze all the data. Data are shown as mean ± S.E.M and as % of baseline to control for unwanted sources of variation. Significance for all tests was assumed at p<0.05.

## 3 Results

### 3.1 Social play

To assess whether a combination of nesting (SH or CN) and early isolation (CTRL or ESI) conditions affected the social domain of the adolescent offspring, we tested the animals in the social play test at PND35 (see timeline in **Figure 1A**). In male rats, the two-way ANOVA revealed a significant effect of CN, ESI and of the CN×ESI interaction on the number of pinning (for details, see **Supplementary Table 1**). *Post-hoc* analyses revealed that the ESI procedure significantly increased the number of pinning only in CN rats and not in SH animals (**Figure 1B**). Conversely, the ANOVA did not show any significant effect of CN, of ESI, and of CN×ESI interaction on the number of pouncing (**Figure 1C**). Similarly, no significant effect of CN, ESI and of CN×ESI interaction on the time spent in general social exploration was found (**Figure 1D**).

In the female offspring, the statistical analysis revealed a significant effect of CN, but no significant effect of ESI or CN×ESI interaction, on the number of pinning (**Figure 1E**). Similarly, the ANOVA revealed a significant effect of CN on the number of pouncing, whereas no significant effect of ESI and of CN×ESI interaction was observed (**Figure 1F**). Regarding the time spent by females in social exploration any significant effect of CN, of ESI, and of CN×ESI interaction was found (**Figure 1G**).

### 3.2 Expression levels of PSD95 and SynCAM 1 in the enriched membrane fraction of mPFC in adolescent rats

We first evaluated the expression of the integral protein of the glutamate synapse, PSD95, in the enriched membrane fraction of rat mPFC in both PND35 male and female rats. Two-way ANOVA revealed a main effect of CN×ESI interaction in both males (**Figure 2A**) and females (**Figure 2C**) (for details, see **Supplementary Table 2**). Further intergroup sub-testing indicated that in both sexes the CN procedure significantly reduced PSD95 expression, whereas the ESI procedure significantly diminished PSD95 expression in SH rats while increasing it in CN rats. We then moved our interest in the analysis of the expression of SynCAM 1, a trans-synaptic organizer contributing to promote excitatory synapse formation (41). In males, two-way ANOVA revealed a main effect of ESI and of CN×ESI interaction (**Figure 2B**). Subdivision by the interactive factors showed no effect of CN procedure per sè, no effect of ESI on SH rats whereas the ESI procedure significantly upregulated SynCAM 1 expression in CN rats. In females, two-way ANOVA revealed a main effect of CN and of the CN×ESI interaction (**Figure 2D**). Lower-order *post-hoc* tests indicated a significant reduction of SynCAM 1 expression as result of CN exposure, whereas the ESI procedure significantly reduced SynCAM 1 expression in SH rats but not in CN rats.

### 3.3 Expression levels of the main NMDA receptor subunits in the enriched membrane fraction of mPFC in adolescent rats

We then analyzed the main NMDA receptor subunits, starting with the measurement of the expression level of the obligatory subunit GluN1.

In males, two-way ANOVA revealed a significant effect of ESI and of CN×ESI interaction for the main NMDA subunit GluN1 (**Figure 3A**) (for details, see **Supplementary Table 2**). The intergroup comparisons revealed a significant increase of GluN1 in the CN+ESI combination. The analysis of GluN2A expression revealed a main effect of the CN× ESI interaction (**Figure 3B**). Further intergroup sub-testing indicated that the CN procedure elicited a significant reduction of GluN2A expression in male rats, whereas the ESI procedure significantly reduced GluN2A expression in SH rats while increasing it in CN rats (**Figure 3B**). Investigation of GluN2B expression in males showed a significant ESI and CN×ESI interaction. *Post-hoc* test indicated a significant increase of this subunit in the SH+ESI combination, with no effect of CN and CN+ESI combination (**Figure 3C**). We then analyzed the ratio GluN2A/GluN2B, considered an index of neuroplasticity. We found a main effect of CN×ESI interaction (**Figure 3D**). In light of this interaction, the analysis of the individual effect revealed that CN procedure significantly reduced the ratio, ESI procedure significantly induced a reduction in SH rats while increasing it in CN rats (**Figure 3D**).

In females, two-way ANOVA revealed a significant effect of CN, of ESI and a CN×ESI interaction on the main subunit GluN1 (**Figure 3E**) (for details, see **Supplementary Table 2**). *Post-hoc* testing revealed that exposure to CN reduced GluN1 expression whereas ESI significantly reduced it only in SH rats (**Figure 3E**). A similar picture was drawn for the GluN2A accessory NMDA subunit, where we observed a significant effect of CN×ESI interaction (**Figure 3F**). Further intergroup sub-testing indicated that CN procedure elicited a reduction of GluN2A levels which were reduced also by ESI in SH-exposed rats, while no further reduction was shown by ESI in CN-exposed rats (**Figure 3F**). The modulation of GluN2B was slightly different from GluN2A. In fact, we observed a significant effect of CN×ESI interaction (**Figure 3G**). Post-hoc analysis revealed a reduction of GluN2B caused by CN per sè and by ESI in SH rats, whereas ESI evoked a significant up-regulation in rats previously exposed to CN (**Figure 3G**). In the GluN2A/GluN2B ratio we found a main effect of CN×ESI interaction (**Figure 3H**). Examining the individual effects, we found that the response to ESI was influenced only in CN-exposed rats, as shown by a significant reduction of GluN2A/GluN2B ratio, whereas no change was caused by the ESI condition in SH rats.

### 3.4 Activation of Synapsin I and αCaMKII in the enriched membrane fraction of mPFC in adolescent rats

Searching for a potential mechanism subserving the effects shown above, we focused on the mechanisms regulating neurotransmitter release. First, we analyzed the activation of Synapsin I, here expressed as the ratio between its phosphorylation at the serine residue 603 and its total levels.

In males, two-way ANOVA revealed a significant effect of CN and of the CN×ESI interaction (**Figure 4A**) (for details, see **Supplementary Table 2**). Post-hoc treatment revealed that the response to ESI was influenced only in CN-exposed rats as shown by a significant up-regulation (**Figure 4A**). Next, we measured the activation of αCaMKII by examining the ratio between pαCaMKII T286 and αCaMKII. We found a main effect of the CN×ESI interaction (**Figure 4B**) (for details, see **Supplementary Table 2**). Subdivision by the interactive factors showed that the pαCaMKII T286/αCaMKII showed a trend (not significant) toward a reduction in male CN rats and the ESI procedure significantly reduced the ratio in SH rats while increasing it in CN rats (**Figure 4B**).

In females, the main effects of CN, ESI and of CN×ESI interaction were observed for pSynapsin I S603/Synapsin I, while for pαCaMKII T286/αCaMKII a main effect was present only for CN and CN×ESI interaction (**Figures 4C, D**). Lower-order post-hoc tests showed that CN per sè reduced both ratios; the response to ESI was reduced in SH rats whereas such ratio either for Synapsin I and αCaMKII was not further modified by the ESI procedure in rats exposed to CN.

### 3.5 Expression levels of PSD95 and SynCAM 1 in the enriched membrane fraction of mPFC in adult rats

Two-way ANOVA revealed a main effect of ESI and a CN×ESI interaction (**Figure 5A**) (for details, see **Supplementary Table 3**) when examining the expression of PSD95 in the mPFC of PND75 male rats. Intergroup sub-testing indicated that the CN procedure significantly reduced PSD95 expression in male rats and that the ESI procedure did not alter PSD95 expression in SH rats while increasing it in CN rats (**Figure 5A**). In females, two-way ANOVA revealed a significant effect of CN, ESI and of CN×ESI interaction (**Figure 5C**). Evaluating the individual effects, we found that ESI evoked a significant increase of PSD95 expression in CN rats whereas no effect was observed in the mPFC of SH rats.

Next, we analyzed the expression of SynCAM 1. In males, two-way ANOVA revealed a main effect of CN×ESI interaction (**Figure 5B**). Post-hoc analysis of the main effects revealed a significant reduction of SynCAM 1 expression following CN procedure per sè; ESI reduced the expression of SynCAM 1 in SH rats while up-regulating it in CN rats (**Figure 5B**). A different situation was observed in females, where two-way ANOVA revealed a significant effects of the CN per sè and of the CN×ESI interaction (**Figure 5D**). In light of such interaction, post-hoc test of the main effects indicated no effect of CN on SynCAM 1 expression, whereas the ESI procedure significantly enhanced SynCAM 1 expression in SH rats while diminishing it in CN rats (**Figure 5D**).

### 3.6 Expression levels of the main NMDA receptor subunits in the enriched membrane fraction of mPFC in adult rats

We next investigated the main NMDA receptor subunits in the mPFC of PND75 rats. Two-way ANOVA revealed a main effect of ESI and a CN×ESI interaction for the main NMDA subunit GluN1 in male rats (**Figure 6A**) (for details, see **Supplementary Table 3**). Post-hoc evaluation of the main effects showed a significant reduction of GluN1 expression in CN and SH+ESI groups, while an enhanced expression of GluN1 in CN+ESI animals (**Figure 6A**). The analysis of GluN2A expression revealed a significant effect of ESI and of CN×ESI interaction (**Figure 6B**). Further intergroup sub-testing indicated that the CN procedure significantly reduced GluN2A expression in male rats, which was significantly increased only after ESI exposure while no further effects were caused by ESI in SH rats (**Figure 6D**). Analysis of GluN2B expression showed a main effect of the CN×ESI interaction (**Figure 6C**) (for details, see **Supplementary Table 3**). Post-hoc test indicated a significant decrease of this subunit by CN; further, we observed that ESI reduced GluN2B expression in SH rats while increasing it in CN rats (**Figure 6C**). The analysis of the ratio GluN2A/GluN2B revealed a significant effect of ESI but no interaction between CN and ESI and, therefore, the data were not further analyzed with post-hoc test (**Figure 6D**).

In females, two-way ANOVA revealed a significant effect of ESI and a CN×ESI interaction on the main subunit GluN1 (**Figure 6E**) and a significant effect of CN, of ESI and a CN×ESI interaction for the GluN2A accessory NMDA subunit (**Figure 6F**) (for details, see **Supplementary Table 3**). Lower-order post-hoc tests were carried out and we found that CN reduced GluN1 and GluN2A expression which were also reduced by ESI in SH-exposed rats, but no further reduction was shown by ESI in CN-exposed rats (**Figures 6E, F**). Analysis of GluN2B expression revealed a main effect of CN, of ESI and a CN×ESI interaction (**Figure 6G**). Post-hoc analysis of the main effects revealed a reduction of GluN2B caused by CN, an increase of this receptor subunit caused by ESI in SH rat and no further change by ESI in rats previously exposed to CN (**Figure 6G**). Analysis of the ratio GluN2A/GluN2B in female rats revealed a significant effect of CN, ESI and of CN×ESI interaction (**Figure 6H**). Examining the individual effects, we found that the response to ESI was influenced only in SH-exposed rats as shown by a significant reduction, whereas no change was caused by ESI in CN rats (**Figure 6H**).

### 3.7 Activation of Synapsin I and αCaMKII in the enriched membrane fraction of mPFC in adult rats

Our next investigation was the analysis of Synapsin I and αCaMKII, as a potential mechanism to sustain the above-mentioned effects.

In males, two-way ANOVA revealed a significant effect of CN×ESI interaction for the ratio pSynapsin I S603/Synapsin I (**Figure 7A**) (for details, see **Supplementary Table 3**). Intergroup sub-testing indicated that the CN procedure reduced pSynapsin I S603/Synapsin I, whereas the ESI procedure reduced pSynapsin I S603/Synapsin I ratio in SH rats, while increasing it in CN rats (**Figure 7A**). In the ratio between pαCaMKII T286/αCaMKII we found a significant main effect of CN and of CN×ESI interaction (**Figure 7B**) (for details, see **Supplementary Table 3**). Post-hoc evaluations revealed that CN reduced the pαCaMKII T286/αCaMKII ratio while the ESI procedure induced an increase of this ratio in CN- but not in SH-exposed rats (**Figure 7B**).

In females, two-way ANOVA showed a significant effect of CN and a CN×ESI interaction for the main ratio pSynapsin I S603/Synapsin I (**Figure 7C**) (for details, see **Supplementary Table 3**). Post-hoc analysis revealed that CN elicited a reduction in response to ESI, whereas ESI did not cause any alteration in SH rats (**Figure 7C**). Conversely to what observed in males, in females the evaluation of the pαCaMKII Thr286/αCaMKII showed an effect of the CN only, with no significant effect of the interaction between CN and ESI (**Figure 7D**).

## 4 Discussion

This study examined the interaction between housing conditions (CN, SH) and environmental manipulations (ESI, CTRL), in the two sexes at two different ages (adolescence, adulthood), and showed that CN primes the glutamate synapse in the rat mPFC of ESI-exposed male, but not female, rats, revealing a sex-dependent effect. From a metaplastic standpoint, it appears that CN plays a permissive, neuroplastic role on the response to ESI in males only. This molecular effect may be functionally relevant since housing in a CN condition disinhibits social play behavior, i.e., a highly pleasurable, rewarding activity critical for a correct social development (42) and it requires the functional integrity of the PFC (19, 43), only in ESI-exposed male rats.

### 4.1 Social play behavior

Pinning (the most characteristic and rewarding component of social play behavior), but not pouncing frequency or time spent in exploration, was significantly more pronounced in males exposed to the CN+ESI combination than in the other experimental groups. Pouncing is a play-soliciting behavior, while pinning is the response to such solicitation (37). Thus, male rats from the CN+ESI group were more responsive to play solicitation than rats from the other experimental groups, i.e., they more frequently responded to play solicitation by pinning the play partner. Conversely, in the female offspring, CN induced a significant increase in the frequency of pinning and pouncing, although no significant effect of ESI or CN×ESI interaction was found. These results are in line with previous studies showing that post-weaning environmental enrichment in rats counteracts the deleterious effects of prenatal stress on social play behavior (44) and with studies showing that maternal exposure to environmental enrichment before and during gestation increases social play behavior in a sex-dependent manner (45). Thus, the possibility exists that the CN condition shapes a distinct maturational trajectory of cortical excitatory synaptic inputs that undergo important remodeling during adolescence and are required for social play (46), in a sex-dependent manner. Importantly, as reported previously (35), pups reared in the CN condition emitted a lower number of USV when isolated from the nest at PND9, whereas no changes were observed in females, confirming the existence of sex-dependent differences in emotionality since the very first phases of life. Interestingly, these behavioral data are the first indication that male pups reared in a socially enriched environment (i.e., CN), known to represent a naturalistic setting able to induce stress resilience (25), might better cope to subsequent challenging events, as suggested by their reduced USV at PND9 and increased pinning during adolescence (47, 48).

### 4.2 Glutamate synapse in adolescent animals

At PND35, males and females respond equally in terms of structural (re)organization of the glutamatergic synapse in the mPFC, as PSD95 levels in SH+ESI and CN+CTRL groups were reduced to a similar extent in the two sexes, suggesting a structural destabilization of the synapse. Yet, when ESI was applied in CN-reared rats, PSD95 levels were normalized, restoring the responsivity and stability of the postsynaptic terminal in both sexes. A different picture emerged from the analysis of the expression of SynCAM 1, a protein essential for the proper activity of glutamatergic terminals that at presynaptic level governs the structural maturation of excitatory synapses, while at post-synaptic level it recruits glutamate receptors (49). A chief finding was a sex-dependent modulation exerted by CN and ESI alone, which decreased protein expression in females but not in males, and by the CN+ESI combination that increased it in males but not in females. This sex-dependent maturation of the synapse suggests that female rats may be more vulnerable to the effects of social isolation early in life despite the social rearing enrichment, whereas CN may confer resilience to ESI in male rats by promoting both SynCAM 1 and PSD95 expression.

Exposure to CN reduced NMDA receptor subunit expression in adolescent females but not in males, with the only exception of GluN2A. In male SH rats, ESI determined an up-regulation of GluN2B and a down-regulation of GluN2A leading to a reduced GluN2A/GluN2B ratio, which can be considered as an index of maladaptive plasticity, i.e., a switch induced by ESI between the GluN2A and GluN2B subunits, which is opposite to the physiological maturation of synapses normally observed during brain development (50). Finding that ESI has blocked the physiological switch in SH but not in CN male rats further highlights the positive, sensitizing effect played by CN in males. Interestingly, CN+ESI caused an up-regulation of GluN1 and GluN2A in males and of GluN2B in females, in which GluN1 was instead down-regulated, suggesting that the CN+ESI combination might have potentiated the glutamate transmission in males, as also evinced by the increased GluN2A/GluN2B ratio. The hypothesis that, in the mPFC of male rats, CN favors the maturation of synapses in response to ESI is supported by the increased expression of GluN1, SynCAM 1 and PSD95 and by the physiological switch of GluN2A (increased) over GluN2B (unchanged). In females, conversely, a reduction of the cortical expression of GluN1 and GluN2A receptor subunits paralleled by increased expression of GluN2B and a reduced ratio GluN2A/GluN2B in response to CN+ESI is indicative of reduced and maladaptive glutamate postsynaptic activity.

To ascertain whether these results could be due to alterations in the release of neurotransmitters, we investigated the activation of Synapsin I, a protein critical for neurotransmitter release (51) and of αCaMKII, known to phosphorylate and activate Synapsin I (52). Notably, the CN+ESI combination in males promoted the activation of αCaMKII and Synapsin I, raising the possibility that the potentiation of glutamate neurotransmission could be due, at least partially, to the activation of the machinery subserving glutamate release. It is interesting to point out that exposure of SH males to ESI reduced the activation of αCaMKII and Synapsin I further supporting the possibility that CN has primed the cortical synapse to up-regulate the machinery subserving the neurotransmitter release. Notably, despite CN females displayed a reduced activation of αCaMKII and Synapsin I, they were not able to activate these molecules following exposure to ESI.

### 4.3 Glutamate synapse in adult animals

Sex difference in the glutamatergic response to early life adversity was readily apparent also during adulthood. In fact, in the mPFC of CN+ESI male rats we found an increased expression of PSD95, SynCAM 1, Synapsin I and GluN1; in the same brain region of females, only PSD95 was upregulated whereas SynCAM 1, Synapsin I and GluN1 were significantly reduced. Notably, age-related differences were observed in the accessory subunits of NMDA receptors as, in males, CN reduced both GluN2A and GluN2B expression whereas ESI reduced GluN1 and GluN2B expression. In agreement with these results, the GluN2A/GluN2B ratio was not altered in CN males whereas it was increased following ESI regardless of the rearing condition. Thus, it appears that in parallel to the long-term influence induced by CN that sensitizes the response to ESI till adulthood, SH rats recover the ability to shape the composition of the NMDA receptor subunits following ESI only in adulthood. Age-dependent differences were found in females as well, as the GluN2A/GluN2B ratio in CN rats was unchanged as result of a reduction of both GluN2A and GluN2B, suggesting the lack of the switch between these two subunits, indicative of a generalized depotentiation of the glutamate synapse (as also confirmed by the reduction of GluN1). SH+ESI females showed heightened vulnerability as the GluN2A/GluN2B ratio was reduced as result of diminished GluN2A and increased GluN2B expression, coupled with a significant decrease of the mandatory GluN1 subunit. This suggests that ESI is sufficient to induce a long-term depotentiation of the glutamate synapse in adulthood, likely through the involvement of Synapsin I, while the modulation of αCaMKII activation seems to be not critical for the effects observed in adulthood.

Altogether, these findings led us to hypothesize that CN can represent an initial priming event that induces molecular changes at the glutamate synapse that can subsequently modulate plasticity induced by ESI; in other words, we suggest that CN has an overall metaplastic effect on the glutamate synapse. Interestingly, this possibility is in line with our recent observations showing that enriched environment (53) or ketamine (54, 55) shape the glutamate synapse in a metaplastic way.

In support to this, the molecular changes in both adolescent and adult CN+ESI males suggest that the recruiting of the NMDA receptor subunits, together with increased expression/activation of PSD95, SynCAM 1, Synapsin I and αCaMKII, may subserve the potentiation of glutamatergic neurotransmission and lead to the ability of male CN rats to cope with ESI. Conversely, the de-recruiting of NMDA receptors from active synaptic zones in both adolescent and adult CN+ESI females, together with reduced expression/activation of the above-mentioned mechanisms, may lead to a depotentiation of glutamate transmission that may underlie the vulnerability of female CN rats to ESI.

### 4.4 Limitations

We are aware that this study is not avoid of limitations. Indeed, electrophysiological validation would narrow down some translational readouts. We focused on mPFC as a brain area critically involved in social play behavior and glutamatergic signaling; yet other brain areas, such as the hippocampus, nucleus accumbens, amygdala, will need to be investigated to understand the general impact of CN, ESI, and their combination on brain development. Similarly, other aspects of the glutamate transmission require future investigation, as Synapsin I and αCaMKII are only a part of the whole mechanism through which glutamate homeostasis modulation occurs in the brain. Last, as early life adverse events, such as maternal separation, produce opposite effects on the mother-infant dyad, according to time of exposure and duration (56), it would be interesting to explore the impact of the ESI protocol on the mother-offspring interaction.

## 5 Conclusion

Our data suggest that, following ESI, the synapse in the mPFC of male rats reared in the CN condition becomes more adaptable, potentially lowering the threshold for synaptic changes to occur when faced with subsequent challenges. In contrast, in females the synapses become less adaptable, lacking the metaplastic mechanism observed in males, which can lead to dysfunction in response to similar future environmental challenges. Learning how CN selectively produces such effects and modulates the response to adverse situations during a critical window of brain development could guide future investigation into sex-dependent mechanisms underlying the response to adverse environmental situations, thus allowing for more targeted treatments of emotionality-related disorders. Indeed, the CN model in rodents holds significant translational value for clinical applications, allowing to study how social support systems influence mental health outcomes, particularly regarding conditions like depression and anxiety, where social isolation plays a crucial role. Through observing rodents in a CN environment, it is possible to explore how social interactions and support affect stress resilience, emotional regulation, and social behavior.

## Data availability statement

The raw data supporting the conclusions of this article will be made available by the authors, without undue reservation.

## Ethics statement

The animal study was approved by Italian Ministry of Health (authorization n. 612/2020-PR). The study was conducted in accordance with the local legislation and institutional requirements.

## Author contributions

FM: Formal analysis, Methodology, Supervision, Validation, Writing – original draft, Writing – review & editing, Investigation, Visualization. BR: Investigation, Writing – original draft, Writing – review & editing. GT: Investigation, Writing – original draft, Writing – review & editing. VB: Formal Analysis, Investigation, Methodology, Supervision, Visualization, Writing – original draft, Writing – review & editing. MD: Formal analysis, Methodology, Writing – review & editing. LR: Investigation, Writing – original draft, Writing – review & editing. SC: Validation, Writing – original draft, Writing – review & editing. RC: Funding acquisition, Validation, Writing – original draft, Writing – review & editing. LF: Funding acquisition, Validation, Writing – original draft, Writing – review & editing. PR: Funding acquisition, Validation, Writing – original draft, Writing – review & editing. FF: Conceptualization, Funding acquisition, Validation, Writing – original draft, Writing – review & editing. VT: Funding acquisition, Methodology, Supervision, Validation, Writing – original draft, Writing – review & editing. LC: Conceptualization, Formal analysis, Funding acquisition, Methodology, Project administration, Supervision, Validation, Writing – original draft, Writing – review & editing.
