# Supplementary material for: Communal nesting shapes the sex-dependent glutamatergic response to early life stress in the rat prefrontal cortex
Source: Front Psychiatry. 2024 May 21;15:1406687. doi: 10.3389/fpsyt.2024.1406687 (PMC11148342; doi:10.3389/fpsyt.2024.1406687)
Supplement: Supplementary file 1 [file DataSheet_1.docx]

Supplementary Material

# Supplementary methods

**Preparation of subcellular fractions**

Prefrontal cortex tissues were homogenized in a glass–glass potter using a cold buffer containing 0.32 M sucrose, 1 mM Hepes solution, 0.1 mM EGTA, 0.1 mM PMSF, pH = 7.4, in presence of a complete set of protease inhibitors and a phosphatase inhibitor cocktail. Each homogenized tissue was first centrifuged at 800 *g* for 10 min. The resulting supernatant was centrifuged at 9,000 *g* for 15 min to obtain the pellet corresponding to the crude synaptosomal fraction, which was resuspended in a buffer containing 20 mM HEPES, 0.1 mM dithiothreitol, and 0.1 mM EGTA, in presence of a complete set of protease and phosphatase inhibitors cocktail.

# Supplementary Figures and Tables

## Supplementary Figures

**Supplementary Figure 1.** Cropped immunoblot related to the expression levels of GluN2A, GluN1, PSD95, pSynapsin I S603, Synapsin I, SynCAM 1, pαCaMKII T286, αCaMKII and β-actin measured in the membrane fraction of mPFC of SH and CN adolescent (PND 35) male rats exposed or not to Early Social Isolation (ESI) presented in **figure 2**, **3** and **4**.

**Supplementary figure 2**. Cropped immunoblot related to the expression levels of GluN2B and β-actin measured in the crude membrane fraction of mPFC of SH and CN adolescent (PND 35) male rats exposed or not to Early Social Isolation (ESI) presented in **figure 3**.

**Supplementary figure 3**. Cropped immunoblot related to the expression levels of GluN2A, GluN2B, GluN1, PSD95, pSynapsin I S603, Synapsin I, SynCAM 1, pαCaMKII T286, αCaMKII and β-actin measured in the crude membrane fraction of mPFC of SH and CN adolescent (PND 35) female rats exposed or not to Early Social Isolation (ESI) presented in **figure 2**, **3** and **4**.

**Supplementary figure 4**. Cropped immunoblot related to the expression levels of GluN2B, GluN1, PSD95, pSynapsin I S603, Synapsin I, SynCAM 1, pαCaMKII T286, αCaMKII and β-actin measured in the crude membrane fraction of mPFC of SH and CN adult (PND 75) male rats exposed or not to Early Social Isolation (ESI) presented in **figure 5**, **6** and **7**.

**Supplementary figure 5**. Cropped immunoblot related to the expression levels of GluN2A and β-actin measured in the crude membrane fraction of mPFC of SH and CN adult (PND 75) male rats exposed or not to Early Social Isolation (ESI) presented in **figure 5**.

**Supplementary figure 6**. Cropped immunoblot related to the expression levels of GluN2A, GluN1, PSD95, pαCaMKII T286, αCaMKII and β-actin measured in the crude membrane fraction of mPFC of SH and CN adult (PND 75) female rats exposed or not to Early Social Isolation (ESI) presented in **figure 5,** **6** and **7**.

**Supplementary figure 7**. Cropped immunoblot related to the expression levels of GluN2B, pSynapsin I S603, Synapsin I, SynCAM 1 and β-actin measured in the crude membrane fraction of mPFC of SH and CN adult (PND 75) female rats exposed or not to Early Social Isolation (ESI) presented in **figure 5, 6** and **7**.

**Supplementary figure 8**. Cropped immunoblot run as duplicate related to the expression levels of GluN2A, GluN2B, GluN1, PSD95, pSynapsin I S603, Synapsin I, SynCAM 1, pαCaMKII T286, αCaMKII and b-actin measured in the membrane fraction of mPFC of SH and CN adolescent (PND 35) male rats exposed or not to Early Social Isolation (ESI) presented in **figure 2**, **3** and **4**.


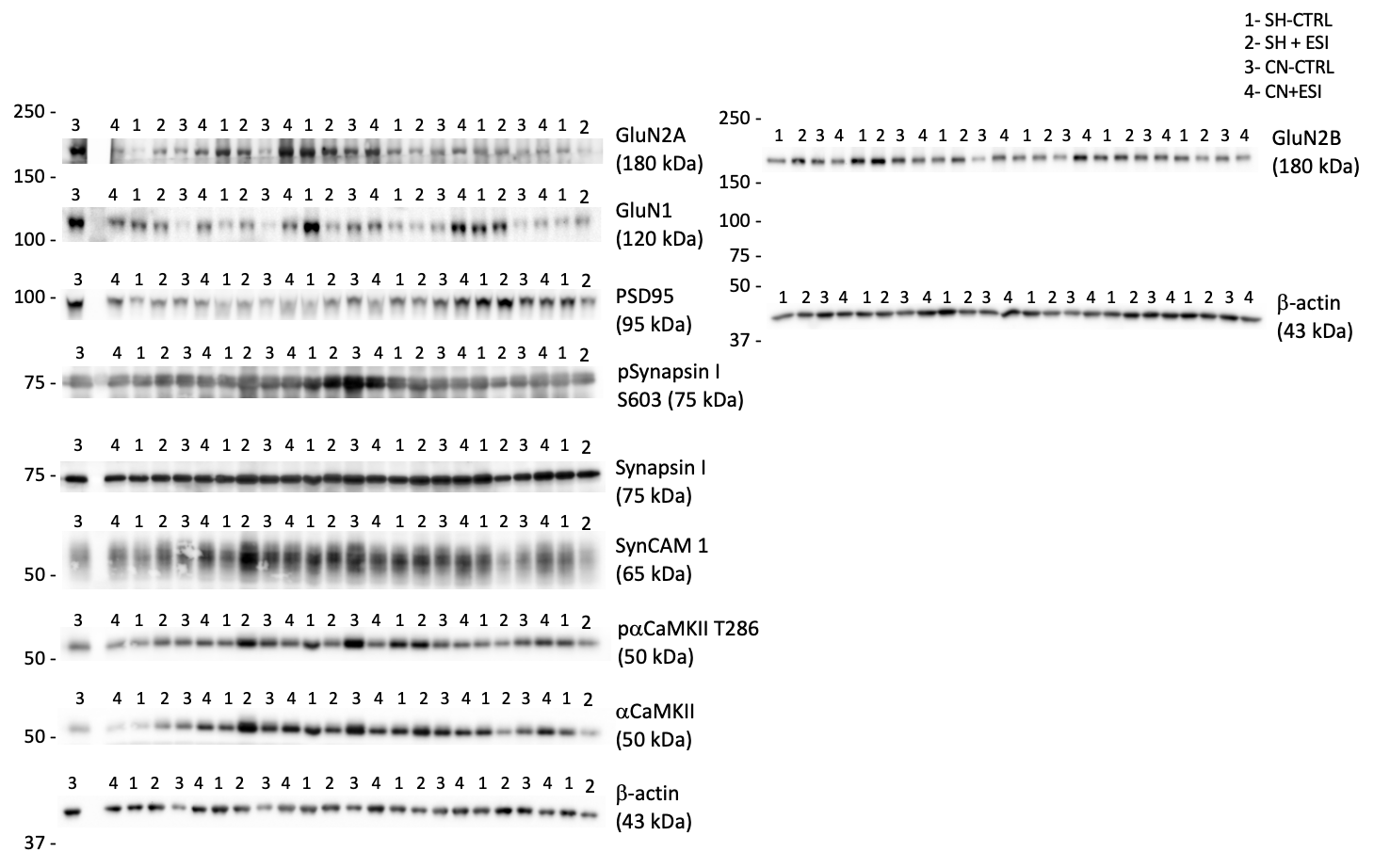


**Supplementary figure 9**. Cropped immunoblot run as duplicate related to the expression levels of GluN2A, GluN2B, GluN1, PSD95, pSynapsin I S603, Synapsin I, SynCAM 1, pαCaMKII T286, αCaMKII and b-actin measured in the crude membrane fraction of mPFC of SH and CN adolescent (PND 35) female rats exposed or not to Early Social Isolation (ESI) presented in **figure 2**, **3** and **4**.


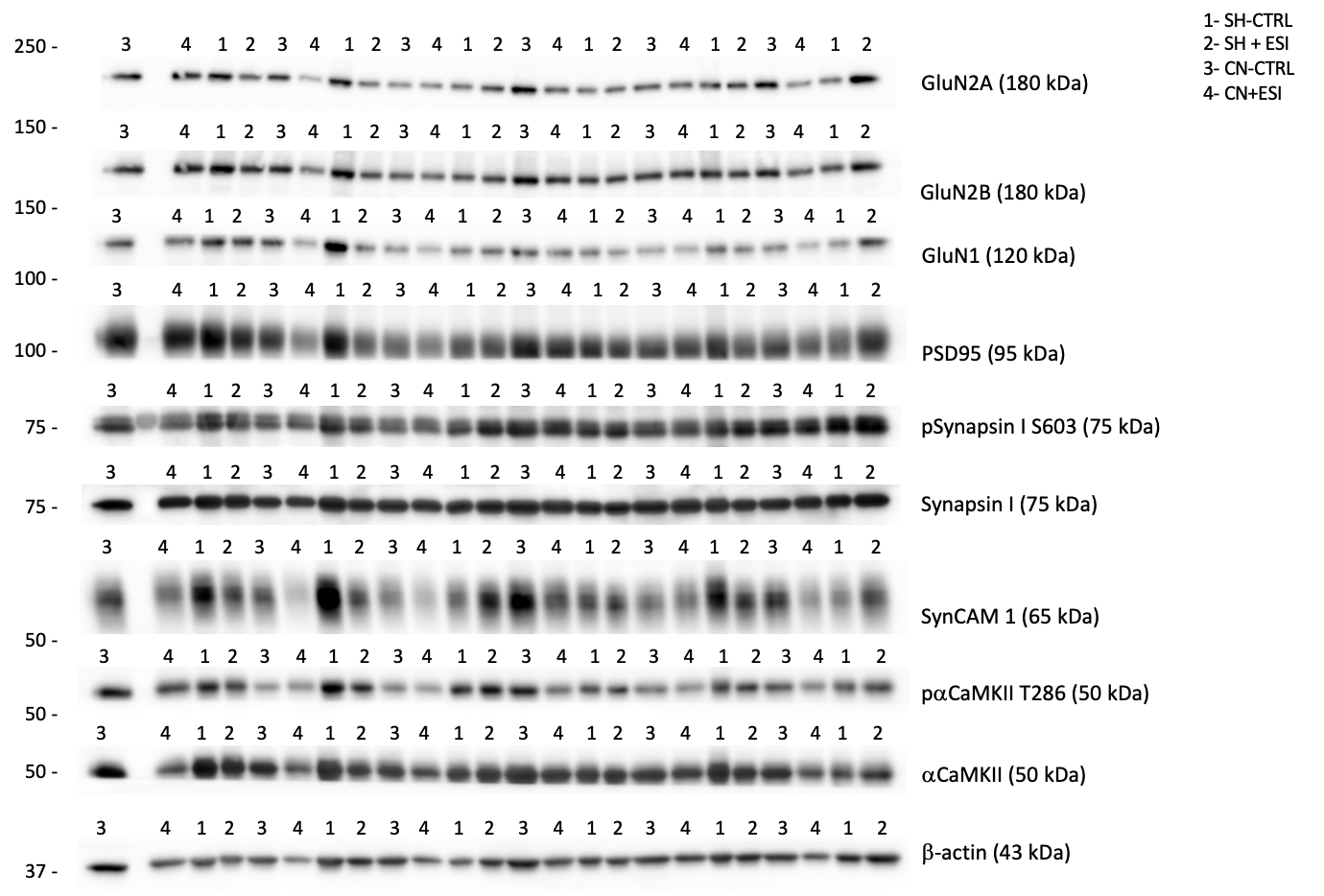


**Supplementary figure 10**. Cropped immunoblot run as duplicate related to the expression levels of GluN2A, GluN2B, GluN1, PSD95, pSynapsin I S603, Synapsin I, SynCAM 1, pαCaMKII T286, αCaMKII and b-actin measured in the membrane fraction of mPFC of SH and CN adult (PND 75) male rats exposed or not to Early Social Isolation (ESI) presented in **figures 5, 6** and **7**.


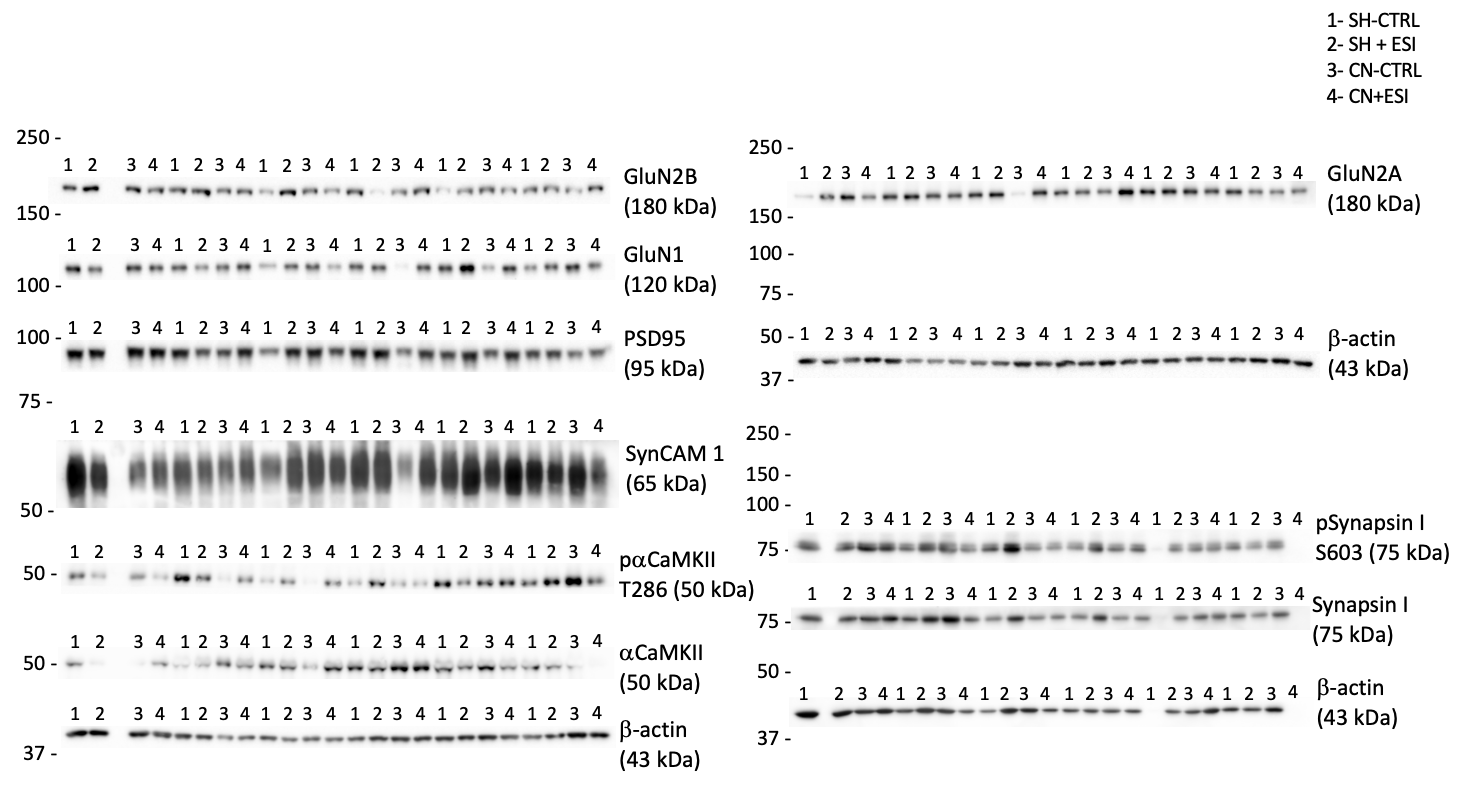


**Supplementary figure 11**. Cropped immunoblot run as duplicates related to the expression levels of GluN2A, GluN2B, GluN1, PSD95, pSynapsin I S603, Synapsin I, SynCAM 1, pαCaMKII T286, αCaMKII and b-actin measured in the membrane fraction of mPFC of SH and CN adult (PND 75) female rats exposed or not to Early Social Isolation (ESI) presented in **figures 5, 6** and **7**.

## Supplementary tables

**Supplementary Table 1** Detailed F values, degrees of freedom and p values relative to two-way ANOVA analyses of social play behaviors measured as number of pinning, number of pouncing and social exploration time of SH+CTRL, SH+ESI, CN+CTRL, CN+ESI male and female rats at post-natal day 35 and presented in figure 1. SH, Standard Housing; CN, Communal Nesting; CTRL, controls no ESI; ESI, Early Social Isolation.

|  |  | Males | | Females | | |
| --- | --- | --- | --- | --- | --- | --- |
| **Two-way ANOVA** | | **F values** | **P value** | **F values** | **P value** |  |
| Pinning | Interaction | F (1, 24) = 4,538 | P=0,0436 | F (1, 25) = 0,006501 | P=0,9364 |  |
|  | Communal Nesting | F (1, 24) = 5,914 | P=0,0229 | F (1, 25) = 6,467 | P=0,0176 |  |
|  | Early Social Isolation | F (1, 24) = 7,066 | P=0,0138 | F (1, 25) = 0,01543 | P=0,9021 |  |
| Pouncing | Interaction | F (1, 24) = 2,002 | P=0,1699 | F (1, 25) = 0,1671 | P=0,6862 |  |
|  | Communal Nesting | F (1, 24) = 3,870 | P=0,0608 | F (1, 25) = 10,78 | P=0,0030 |  |
|  | Early Social Isolation | F (1, 24) = 0,6301 | P=0,4351 | F (1, 25) = 0,3736 | P=0,5466 |  |
| Social exploration | Interaction | F (1, 24) = 0,4218 | P=0,5222 | F (1, 25) = 0,1675 | P=0,6858 |  |
|  | Communal Nesting | F (1, 24) = 3,007 | P=0,0957 | F (1, 25) = 0,3390 | P=0,5656 |  |
|  | Early Social Isolation | F (1, 24) = 1,430 | P=0,2435 | F (1, 25) = 1,370 | P=0,2529 |  |

**Supplementary Table 2** Detailed F values, degrees of freedom and p values relative to two-way ANOVA analyses of protein levels in the prefrontal cortex of SH+CTRL, SH+ESI, CN+CTRL, CN+ESI male and female rats at post-natal day 35 and presented in figures 2,3 and 4. SH, Standard Housing; CN, Communal Nesting; CTRL, controls no ESI; ESI, Early Social Isolation.

|  |  | Males | | | | Females | |
| --- | --- | --- | --- | --- | --- | --- | --- |
| **Two-way ANOVA** | | | **F values** | | **P value** | **F values** | **P value** |
| PSD95 | Interaction | F (1, 20) = 28,68 | | P<0,0001 | | F (1, 20) = 26,95 | P<0,0001 |
|  | Communal Nesting | F (1, 20) = 0,04486 | | P=0,8344 | | F (1, 20) = 1,653 | P=0,2133 |
|  | Early Social Isolation | F (1, 20) = 1,785 | | P=0,1965 | | F (1, 20) = 0,5750 | P=0,4571 |
| SynCAM 1 | Interaction | F (1, 20) = 5,348 | | P=0,0315 | | F (1, 20) = 7,959 | P=0,0106 |
|  | Communal Nesting | F (1, 20) = 2,931 | | P=0,1023 | | F (1, 20) = 5,541 | P=0,0289 |
|  | Early Social Isolation | F (1, 20) = 9,823 | | P=0,0052 | | F (1, 20) = 2,933 | P=0,1022 |
| GluN1 | Interaction | F (1, 20) = 9,031 | | P=0,0070 | | F (1, 20) = 4,529 | P=0,0459 |
|  | Communal Nesting | F (1, 20) = 0,2026 | | P=0,6575 | | F (1, 20) = 13,32 | P=0,0016 |
|  | Early Social Isolation | F (1, 20) = 6,205 | | P=0,0216 | | F (1, 20) = 15,32 | P=0,0009 |
| GluN2A | Interaction | F (1, 19) = 30,31 | | P<0,0001 | | F (1, 20) = 7,115 | P=0,0148 |
|  | Communal Nesting | F (1, 19) = 0,8125 | | P=0,3787 | | F (1, 20) = 2,516 | P=0,1284 |
|  | Early Social Isolation | F (1, 19) = 0,6939 | | P=0,4152 | | F (1, 20) = 1,949 | P=0,1780 |
| GluN2B | Interaction | F (1, 20) = 6,943 | | P=0,0159 | | F (1, 20) = 54,22 | P<0,0001 |
|  | Communal Nesting | F (1, 20) = 2,933 | | P=0,1022 | | F (1, 20) = 0,5091 | P=0,4838 |
|  | Early Social Isolation | F (1, 20) = 6,743 | | P=0,0172 | | F (1, 20) = 1,423 | P=0,2468 |
| GluN2A/GluN2B | Interaction | F (1, 19) = 29,24 | | P<0,0001 | | F (1, 20) = 4,765 | P=0,0411 |
|  | Communal Nesting | F (1, 19) = 1,218 | | P=0,2835 | | F (1, 20) = 2,795 | P=0,1101 |
|  | Early Social Isolation | F (1, 19) = 0,5851 | | P=0,4537 | | F (1, 20) = 3,247 | P=0,0866 |
| pSyn I/Syn I | Interaction | F (1, 20) = 12,57 | | P=0,0020 | | F (1, 20) = 11,20 | P=0,0032 |
|  | Communal Nesting | F (1, 20) = 4,686 | | P=0,0427 | | F (1, 20) = 11,67 | P=0,0027 |
|  | Early Social Isolation | F (1, 20) = 0,3896 | | P=0,5396 | | F (1, 20) = 5,032 | P=0,0364 |
| pαCaMKII/αCaMKII | Interaction | F (1, 20) = 26,38 | | P<0,0001 | | F (1, 20) = 4,420 | P=0,0484 |
|  | Communal Nesting | F (1, 20) = 3,884 | | P=0,0627 | | F (1, 20) = 14,65 | P=0,0011 |
|  | Early Social Isolation | F (1, 20) = 0,006286 | | P=0,9376 | | F (1, 20) = 3,723 | P=0,0680 |

**Supplementary Table 3** Detailed F values, degrees of freedom and p values relative to two-way ANOVA analyses of protein levels in the prefrontal cortex of SH+CTRL, SH+ESI, CN+CTRL, CN+ESI male and female rats at post-natal day 75 and presented in figures 5,6 and 7. SH, Standard Housing; CN, Communal Nesting; CTRL, controls no ESI; ESI, Early Social Isolation.

|  |  | Males | | Females | |  |
| --- | --- | --- | --- | --- | --- | --- |
| **Two-way ANOVA** | | **F values** | **P value** | **F values** | **P value** | |
| PSD95 | Interaction | F (1, 20) = 18,33 | P=0,0004 | F (1, 20) = 19,91 | P=0,0002 | |
|  | Communal Nesting | F (1, 20) = 0,4624 | P=0,5043 | F (1, 20) = 19,50 | P=0,0003 | |
|  | Early Social Isolation | F (1, 20) = 26,14 | P<0,0001 | F (1, 20) = 20,90 | P=0,0002 | |
| SynCAM 1 | Interaction | F (1, 19) = 25,40 | P<0,0001 | F (1, 20) = 21,01 | P=0,0002 | |
|  | Communal Nesting | F (1, 19) = 0,01487 | P=0,9042 | F (1, 20) = 27,86 | P<0,0001 | |
|  | Early Social Isolation | F (1, 19) = 1,162 | P=0,2946 | F (1, 20) = 0,07738 | P=0,7837 | |
| GluN1 | Interaction | F (1, 20) = 67,54 | P<0,0001 | F (1, 20) = 5,853 | P=0,0252 | |
|  | Communal Nesting | F (1, 20) = 2,819 | P=0,1087 | F (1, 20) = 3,696 | P=0,0689 | |
|  | Early Social Isolation | F (1, 20) = 10,14 | P=0,0047 | F (1, 20) = 5,839 | P=0,0254 | |
| GluN2A | Interaction | F (1, 20) = 9,636 | P=0,0056 | F (1, 20) = 5,505 | P=0,0294 | |
|  | Communal Nesting | F (1, 20) = 1,272 | P=0,2727 | F (1, 20) = 26,59 | P<0,0001 | |
|  | Early Social Isolation | F (1, 20) = 18,52 | P=0,0003 | F (1, 20) = 13,95 | P=0,0013 | |
| GluN2B | Interaction | F (1, 20) = 24,12 | P<0,0001 | F (1, 20) = 4,497 | P=0,0466 | |
|  | Communal Nesting | F (1, 20) = 2,220 | P=0,1518 | F (1, 20) = 111,6 | P<0,0001 | |
|  | Early Social Isolation | F (1, 20) = 0,01384 | P=0,9075 | F (1, 20) = 6,105 | P=0,0226 | |
| GluN2A/GluN2B | Interaction | F (1, 20) = 0,3631 | P=0,5536 | F (1, 20) = 4,649 | P=0,0434 | |
|  | Communal Nesting | F (1, 20) = 0,2118 | P=0,6503 | F (1, 20) = 8,342 | P=0,0091 | |
|  | Early Social Isolation | F (1, 20) = 22,21 | P=0,0001 | F (1, 20) = 12,59 | P=0,0020 | |
| pSyn I/Syn I | Interaction | F (1, 20) = 21,31 | P=0,0002 | F (1, 20) = 6,438 | P=0,0196 | |
|  | Communal Nesting | F (1, 20) = 5,974e-005 | P=0,9939 | F (1, 20) = 5,800 | P=0,0258 | |
|  | Early Social Isolation | F (1, 20) = 0,2140 | P=0,6486 | F (1, 20) = 4,173 | P=0,0545 | |
| pαCaMKII/αCaMKII | Interaction | F (1, 20) = 12,39 | P=0,0022 | F (1, 20) = 0,07623 | P=0,7853 | |
|  | Communal Nesting | F (1, 20) = 0,4122 | P=0,5281 | F (1, 20) = 4,711 | P=0,0422 | |
|  | Early Social Isolation | F (1, 20) = 19,93 | P=0,0002 | F (1, 20) = 0,1336 | P=0,7185 | |

**
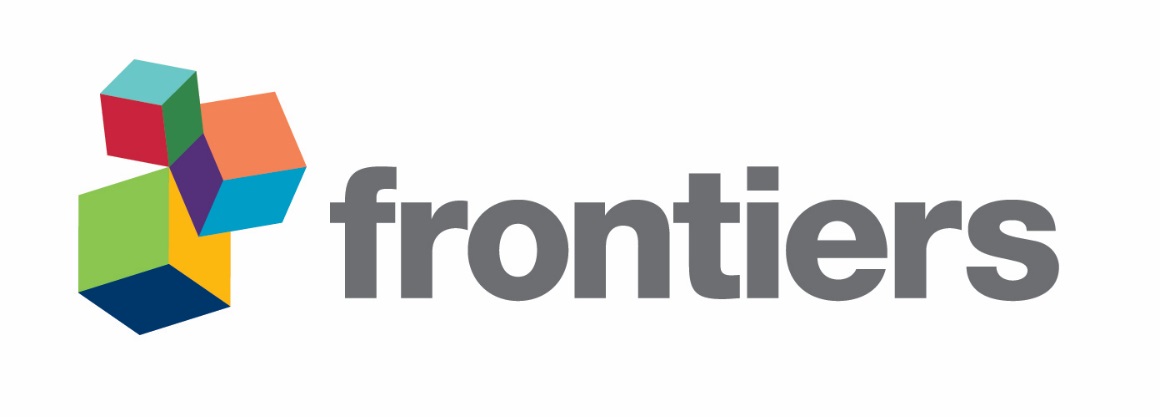
**
